# Medical Politics in Yorkshire

**Published:** 1895-08-03

**Authors:** 


The Hospital

An Institutional Journal of

The Medical Sciences and Hospital Administration, with

Vol. XVIII.—No. 462.] AN EXTRA NURSING SUPPLEMENT. [Aug. 3, 1895.

### Medical Politics in Yorkshire.

At Bradford, during the recent Parliamentary election, an attempt was made by some of the leading medical practitioners in the town to get the candidates to understand certain medical "grievances," and to promise their help towards the legislative removal of those grievances. There were nine candidates for Parliamentary honours, and the committee acting on behalf of the local medical profession, addressed itself to them all. The answers given showed clearly that so far as the Radical politicians were concerned there was a marked disposition to regard medical grievances as "class" grievances, and, therefore, as unworthy of attention. On the other hand, the Conservatives and Liberal Unionists spoke their minds quite definitely, and generally agreed that the "grievances" complained of by the doctors were "real," and ought to be dealt with by Parliament.

About the "reality" of the grievances brought before their candidates by the Bradford doctors there can be no doubt whatever. As to their substantiality, the doctors themselves, who feel the pinch of the shoe, are quite convinced, whatever Parliamentary candidates may feel on the subject. Of course, we are aware that all classes, as well as those who are supposed to belong to the "masses" rather than the classes, have plenty of grievances to complain of. But there is a peculiarity about the particular medical grievances brought forward by the Bradford doctors which puts them into a category quite separate and distinct from the ordinary grievances of which the public, including doctors, are always ready to complain. The peculiarity of the particular medical grievances put forward at Bradford is that they are entirely un-English. They are absolutely opposed to the whole spirit and tendency of our national constitution. The whole of our history for the past thousand years has shown a continuous effort on the part of Englishmen to throw off, and stamp out, and extinguish, just such grievances as these. They are, in fact, grievances which contravene and trample upon the most elementary rights of the British subject, the most rudimentary principles of all political liberty.

Here are two of the questions which were sent round in printed circulars to the candidates by the Bradford Medical Committee: "Do you consider it in accordance with justice, and with the spirit of British legislation, that the State should exact forced labour from any class of its citizens?" "Do you think it right that the State should compel medical men to render important public services, for which, notwithstanding, it absolutely declines to make payment?" Of course every medical man who has a spark of political vitality in him knows what those questions are aimed at. The State, for example, compels a medical man attending on a patient to give, in the event of death, a certificate of the cause of death, age, sex, and other particulars, for which, nevertheless, it positively refuses payment, even of the smallest amount. Similarly it exacts from the family doctor, that is, from every family doctor, not merely from the parish doctor, certificates of the successful or non-successful performance of vaccination in all cases he may vaccinate, also without payment. These unremunerated services the State has exacted from our unwilling and justly indignant profession for many years. Quite recently it has added to these duties the notification, under severe penalties, of every form of contagious and infectious disease. This latter is a very onerous service indeed, and in the case of epidemics such as those of scarlatina and the like, a service which may not seldom prove the last straw in his daily task that may make the overworked practitioner break down.

Now on the ground of high political "right" it may be successfully argued that the State has no claim to exact such services from medical men at all. If the medical profession were to insist upon its strict constitutional "rights," and to put its case competently before the legislature, it could successfully maintain a refusal to render any such services whatever as the State insists upon exacting from it. But the doctor, being a man of education, knows quite well that the certification of the cause of death and of successful or unsuccessful vaccination, and the like, are of such paramount importance to the public well-being that he never dreams of standing upon his strict constitutional rights. He gives way, therefore, in the public interest, on the abstract political question of what the Bradford memorialists somewhat quaintly call "forced labour," and permits the legislature, without protest, to make penal enactments compelling him to certify. So far good. But the most amiable of worms, if trodden upon and kicked about with sufficient perseverance, will sometimes turn; and when the State, in addition to its exaction of "forced labour," meanly and stupidly refuses all remuneration of every kind, then the medical worm begins to think whether it cannot evolve itself into a boa constrictor, and show Parliament that it has got some teeth and is minded to bite. In our judgment it ought to bite. As medical men we ought to insist that neither for private persons nor for the body called the State will we render unremunerated service, except of our own free will. A great deal of work we are constantly doing without payment, both for the State and for private individuals; and we do it willingly, as recognising the position of "humanity" which, by virtue of our knowledge, we hold among our fellow men. But when the State lays down laws which tell us that we shall, whether we like it or not, render it certain services, and that without payment, then all that is English in us rises up in protest that insists upon being heard. Our answer to such unreasonable and unjust legislation is this, that if we are to be compelled to render services to the State whether we like it or not, our self-respect will not permit us to render such services except upon just and adequate payment. Any other position than this is a deliberate consent upon the part of the medical profession to its own partial enslavement.

